# Dynamic response of a cracked atomic force microscope cantilever used for nanomachining

**DOI:** 10.1186/1556-276X-7-131

**Published:** 2012-02-15

**Authors:** Haw-Long Lee, Win-Jin Chang

**Affiliations:** 1 Department of Mechanical Engineering, Kun Shan University, Tainan 71003, Taiwan

**Keywords:** atomic force microscope, cracked cantilever, nanomachining, vibration response1

## Abstract

The vibration behavior of an atomic force microscope [AFM] cantilever with a crack during the nanomachining process is studied. The cantilever is divided into two segments by the crack, and a rotational spring is used to simulate the crack. The two individual governing equations of transverse vibration for the cracked cantilever can be expressed. However, the corresponding boundary conditions are coupled because of the crack interaction. Analytical expressions for the vibration displacement and natural frequency of the cracked cantilever are obtained. In addition, the effects of crack flexibility, crack location, and tip length on the vibration displacement of the cantilever are analyzed. Results show that the crack occurs in the AFM cantilever that can significantly affect its vibration response.

**PACS: **07.79.Lh; 62.20.mt; 62.25.Jk

## Introduction

Since its invention in 1986 [1], the atomic force microscope [AFM] has become a very powerful tool for studying the surface characteristics of diverse materials on a micro- and nanoscale level [2-4]. In addition, atomic force microscopy can also be applied to nanoscale lithography. AFM-based nanolithography technique has a very high potential for nanofabrication [5-8]. The nanolithography research can be roughly divided into three categories: (1) local electrochemical reactions of silicon and metal, (2) direct atomic and molecular manipulation, and (3) direct nanomachining of the material. The direct nanomachining of material structures has so far been useful for fabricating nanodevices.

The nanomachining technique has been the interest of many researchers. Horng [9] studied the flexural vibration responses of a rectangular AFM cantilever subjected to a cutting force using the modal superposition method. Voigt et al. [10] utilized a chip cantilever system for material processing tasks on the micro- and nanometer scales by the flexural-torsional resonance mode. Recently, Zhu et al. [11] investigated the AFM-based nanometric cutting process of copper using molecular dynamics simulation.

Cracks may occur in the AFM cantilever during the nanomachining experiments or in the fabrication of cantilever [12]. The cantilever with cracks will affect its performance when used. The finite element method and finite difference method can be used to analyze the vibration response of a beam with cracks. For example, Sinha and Friswell [13] investigated the vibration behavior of a free-free beam with a breathing crack using a finite element model. Dorogoy and Banks-Sills [14] utilized a finite difference method to study the effect of contact and friction on the disk specimens containing a crack that are subjected to concentrated loads. Based on our literature survey, however, there has been no investigation on the mechanical properties of an AFM cantilever with cracks. The vibration response of a cantilever is related to the processing quality. In this paper, the vibration behavior of the cracked cantilever during the nanomachining process is studied. An analytical expression of vibration displacement of the cracked cantilever is obtained. Both the finite element and finite difference methods are a numerical method. However, the method adopted in this paper is an analytical model. In general, the solution obtained using the analytical method is more accurate than that of the numerical method. In addition, the effects of crack flexibility and crack location on the displacement are investigated.

## Analysis

An AFM probe is used to machine the specimen, and it is considered as a cantilever beam which has Young's modulus *E*, moment of inertia *I*, density *ρ*, the uniform cross-section *A*, and length *L*. When the machining is in progress, the cantilever tip contacts with the specimen and induces a vertical reaction force, *F_y_*(*t*), and a horizontal reaction force, *F_x_*(*t*), which are a function of time *t*. Assuming that the reaction forces are on the tip end, the product of the horizontal force and the tip length can form the equivalent moment exerted on the cantilever. The cutting system can be modeled as a flexural vibration motion of the cantilever. The motion is a partial differential equation, and its transverse displacement is dependent on time *t *and the spatial coordinate *X*.

**Figure 1:**
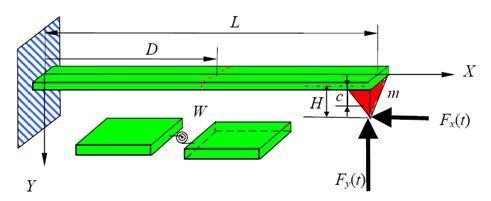
**Schematic diagram of a cracked AFM cantilever nanomachining a sample**. The cutting force is expressed as normal and horizontal forces.

Assume the cantilever has a crack at a distance *D *from the fixed end, and in the vicinity of the crack, there are no the abrupt change of the cross-section. The rotational spring is used to simulate the crack; the cantilever will be divided into two segments by the spring as shown in Figure 1. The spring is assumed to be massless. The cantilever vibration behavior is changed according to the sample properties when the sample surface is in contact with a cantilever tip. The dynamical behavior is complex and highly nonlinear [15]. Simplification is often realized by modeling the cantilever as a linear approximation for the tip-sample interaction forces or a one degree of freedom system [16,17]. This paper investigates the vibration behavior of an AFM cantilever with a crack during the nanomachining process. A relatively large force is generated to perform the machining operation. The effects of van der Waals force, surface stress, electrostatic force, and residual stress are assumed to be negligible. Therefore, the governing equation of transverse vibration of the cantilever with a crack can be expressed by the Euler-Bernoulli equation as follows [18]:

(1) $$EI\frac{\partial^{4}Y_{1}}{\partial X^{4}}+\rho A\frac{\partial^{2}Y_{1}}{\partial t^{2}}=0,\;0\leq X\leq D$$

(2) $$EI\frac{\partial^{4}Y_{2}}{\partial X^{4}}+\rho A\frac{\partial^{2}Y_{2}}{\partial t^{2}}=0,\;D\leq X\leq L,$$

where *X *is the distance along the center of the cantilever, *t *is time, and *Y*_1_(*X,t*) and *Y*_2_(*X, t*) are the transverse displacement of both segments, respectively.

For compatibility of displacement, moment and shear force of for the two adjacent portions of the cantilever can be expressed by the following jump conditions:

(3) $$Y_{1}\left(D,t\right)=Y_{2}\left(D,t\right)$$

(4) $$\frac{\partial^{2}Y_{1}\left(D,t\right)}{\partial X^{2}}=\frac{\partial^{2}Y_{2}\left(D,t\right)}{\partial X^{2}}$$

(5) $$\frac{\partial^{3}Y_{1}\left(D,t\right)}{\partial X^{3}}=\frac{\partial^{3}Y_{2}\left(D,t\right)}{\partial X^{3}}$$

Since, the crack is simulated by a rotational spring; the angular displacement between the two segments can be expressed by the following equation:

(6) $$W\left(\frac{\partial Y_{2}\left(D,t\right)}{\partial X}-\frac{\partial Y_{1}\left(D,t\right)}{\partial X}\right)=EI\frac{\partial^{2}Y_{1}\left(D,t\right)}{\partial X^{2}},$$

where *W *is the rotational spring constant, and *I *stands for the mass moment of inertia.

Finally, the boundary conditions for the AFM cantilever are the following [19]:

(7) $$Y_{1}\left(0,t\right)=0$$

(8) $$\frac{\partial Y_{1}\left(0,t\right)}{\partial X}=0$$

(9) $$EI\frac{\partial^{2}Y_{2}\left(L,t\right)}{\partial X^{2}}=-HF_{x}\left(t\right)-mc^{2}\frac{\partial^{3}Y_{2}\left(L,t\right)}{\partial X\partial t^{2}}$$

(10) $$EI\frac{\partial^{3}Y_{2}\left(L,t\right)}{\partial X^{3}}=F_{y}\left(t\right)+m\frac{\partial^{2}Y_{2}\left(L,t\right)}{\partial t^{2}},$$

where *H *and *m *are the tip height and mass, respectively; *c *is the distance between the lower edge of the cantilever and centroid of the cross section. The boundary condition of the cantilever at *X *= 0 is assumed a fixed end; then, the boundary conditions given by Equations 7 and 8 correspond to conditions of zero displacement and zero slope. The commercial cantilevers are fabricated with a holder providing the base to which the cantilever is suspended. The holder is assumed to be rigid, and the vertical and horizontal reaction forces and fixing moment at the built-in fixed end of the cantilever are to be neglected in the analysis. In addition, the boundary conditions that are given by Equations 9 and 10 correspond to the moment and the force balanced between the beam and a combination of the linear tip-sample stiffness at *X *= *L*, respectively. *F_y _*(*t*) and *F_x _*(*t*) which are both functions of time *t *are denoted the vertical and horizontal cutting force on the sample under the normal and lateral direction, respectively. The first term on right side in Equation 9 represents the moment due to the lateral tip-sample cutting force, and the second term denotes the moment due to the mass moment of inertia of the tip. Similarly, the first term on the right side in Equation 10 is the normal tip-sample interaction force, and the second term is the inertia force of tip mass [19]. The relationship between *F*_*x *_and *F*_*y *_can be expressed as $F_{x}=\frac{2\cot\theta }{\pi }F_{y}$. The relationship is obtained from a geometrical relation for a cone-shape tip, and *θ *is a half-conic angle [20].

The vertical cutting force is assumed to be as follows:

(11) $$F_{y}\left(t\right)=P\sin\left(\text{Ω}t\right),$$

where Ω is the excitation frequency of the cutting force, and *P *is the arbitrary constant.

The dimensionless variables are defined as follows:

(12) $$\begin{array}{c} x=\frac{X}{L},\;v_{1}=\frac{Y_{1}}{L},\;v_{2}=\frac{Y_{2}}{L}\text{, }\alpha =\frac{D}{L},\;h=\frac{H}{L},\;\delta =\frac{c}{L},\;k_{c}=\frac{EI}{WL},\;\varepsilon =\frac{m}{\rho AL},\\ \tau =\frac{t}{\sqrt{\rho AL^{4}/EI}},\;\text{Λ}=\frac{\text{Ω}}{\sqrt{EI/\rho AL^{4}}} \end{array},$$

where *v*_1 _and *v*_2 _are the dimensionless transverse displacement of the two segments; *τ, ε, h*, and *δ *are the dimensionless time, tip mass, length, and centroid of tip, respectively; *k_c _*and α are the dimensionless crack flexibility and crack location, respectively; and Λ is the dimensionless excitation frequency in the transverse direction of the cantilever. Accordingly, the dimensionless vertical and horizontal cutting forces are expressed as follows:

(13) $$F_{y}\left(\tau \right)=f_{y}\sin\left(\text{Λ}\tau \right)\text{ and }F_{x}\left(\tau \right)=\frac{2\cot\theta }{\pi }f_{y}\sin\left(\text{Λ}\tau \right),$$

where $f_{y}=\frac{PL^{2}}{EI}$ are the dimensionless constants relevant to the cutting force in the vertical direction.

The vibration displacements of the two segments can be assumed in the following forms:

(14) $$v_{1}=y_{1}\left(x\right)\sin\left(\text{Λ}\tau \right)\text{ and }v_{2}=y_{2}\left(x\right)\sin\left(\text{Λ}\tau \right).$$

Substituting the harmonic solution given by Equation 14 into Equations 1 to 10 and using the dimensionless variables given by Equation 12, the governing equation and the compatibility and boundary conditions can be simplified to the following dimensionless equations:

(15) $$\frac{d^{4}y_{1}}{dx^{4}}+{\text{Λ}}^{2}y_{1}=0,\;0\leq x\leq \alpha$$

(16) $$\frac{d^{4}y_{2}}{dx^{4}}+{\text{Λ}}^{2}y_{2}=0,\;\alpha \leq x\leq 1$$

(17) $$y_{1}\left(\alpha \right)=y_{2}\left(\alpha \right)$$

(18) $$\frac{d^{2}y_{1}\left(\alpha \right)}{dx^{2}}=\frac{d^{2}y_{2}\left(\alpha \right)}{dx^{2}}$$

(19) $$\frac{d^{3}y_{1}\left(\alpha \right)}{dx^{3}}=\frac{d^{3}y_{2}\left(\alpha \right)}{dx^{3}}$$

(20) $$\frac{dy_{2}\left(\alpha \right)}{dx}-\frac{dy_{1}\left(\alpha \right)}{dx}=k_{c}\frac{d^{2}y_{1}\left(\alpha \right)}{dx^{2}}$$

(21) $$y_{1}\left(0\right)=\frac{dy_{1}\left(0\right)}{dx}=0$$

(22) $$\frac{d^{2}y_{2}\left(1\right)}{dx^{2}}=-hf_{y}\frac{2\cot\theta }{\pi }+{\text{Λ}}^{2}\varepsilon \delta^{2}\frac{dy_{2}\left(1\right)}{dx}$$

(23) $$\frac{\partial^{3}y_{2}\left(1,\tau \right)}{\partial x^{3}}=f_{y}-\varepsilon {\text{Λ}}^{2}y_{2}\left(1\right).$$

The solutions of Equations 15 and 16 can be expressed in the following forms:

(24) $$y_{1}\left(x\right)=B_{1}\sin\left(\gamma x\right)+B_{2}\cos\left(\gamma x\right)+B_{3}\sinh\left(\gamma x\right)+B_{4}\cosh\left(\gamma x\right)$$

(25) $$y_{2}\left(x\right)=B_{5}\sin\left(\gamma x\right)+B_{6}\cos\left(\gamma x\right)+B_{7}\sinh\left(\gamma x\right)+B_{8}\cosh\left(\gamma x\right),$$

where *B*_1_...*B*_8 _are arbitrary constants, *γ *is the wave number, and γ^4 ^= Λ^2^.

Using the compatibility and boundary conditions given in Equations 17 to 23, a 8 × 8 linear system for the AFM cantilever with a crack is obtained:

(26) $$\left[A\right]\left[B\right]=\left[f\right],$$

where

(27) $$\left[B\right]=\left[\begin{array}{c} B_{1}\\ B_{2}\\ B_{3}\\ B_{4}\\ B_{5}\\ B_{6}\\ B_{7}\\ B_{8} \end{array}\right],\;\left[f\right]=\left[\begin{array}{c} 0\\ 0\\ 0\\ 0\\ 0\\ 0\\ -hf_{y}\frac{2\cot\theta }{\pi }\\ f_{y} \end{array}\right],\text{ and}\left[A\right]=[\begin{array}{cccc} \text{s} & \text{c} & \text{sh} & \text{ch}\\ \gamma k_{c}s-c & \gamma k_{c}c+s & -\text{ch}-\gamma k_{c}\text{sh} & -\gamma k_{c}\text{ch}-\text{sh}\\ s & c & -\text{sh} & -\text{ch}\\ c & -s & -\text{ch} & -\text{sh}\\ 1 & 0 & 1 & 0\\ 0 & 1 & 0 & 1\\ 0 & 0 & 0 & 0\\ 0 & 0 & 0 & 0 \end{array}\begin{array}{cccc} -s & -c & -\text{sh} & -\text{ch}\\ c & -s & \text{ch} & \text{sh}\\ -s & -c & \text{sh} & \text{ch}\\ -c & s & \text{ch} & \text{sh}\\ \text{0} & \text{0} & \text{0} & \text{0}\\ \text{0} & \text{0} & \text{0} & \text{0}\\ \xi C-\gamma^{2}S & -\xi S-\gamma^{2}C & \xi \text{Ch}+\gamma^{2}\text{Sh} & \gamma^{2}\text{Ch}+\xi \text{Sh}\\ \eta S-\gamma^{3}C & \eta C+\gamma^{3}S & \gamma^{3}\text{Ch}+\eta \text{Sh} & \eta \text{Ch}+\gamma^{3}\text{Sh} \end{array}],$$

where

(28) $$\begin{array}{c} c=\cos\left(\alpha \gamma \right),\;s=\sin\left(\alpha \gamma \right),\text{ ch}=\cosh\left(\alpha \gamma \right),\text{ sh}=\sinh\left(\alpha \gamma \right),\\ C=\cos\left(\gamma \right),\;S=\sin\left(\gamma \right),\text{ Ch}=\cosh\left(\gamma \right),\text{ Sh}=\sinh\left(\gamma \right), \end{array},$$

$$\xi =-\varepsilon \delta^{2}\gamma^{5}\text{, and }\eta =\varepsilon \gamma^{4}.$$

If the determinant of *A *is set to be zero (i.e., $\left|A\right|=0$), the wave number *γ *of the AFM cantilever with a crack will be solved. Then, the dimensionless free vibration frequency *ω*_*n *_will also be obtained by *ω_n _*= *γ*^2^, while the dimensionless excitation frequency Λ is set as follows:

(29) $$\text{Λ}=r\omega_{n},$$

where *r *is the frequency ratio. The displacement of the cantilever with a crack can be solved from the equation:

(30) $$\left[B\right]={\left[A\right]}^{-1}\left[f\right].$$

If no crack (i.e., *k_c _*= 0) is taken into account in the analysis, the frequency equation can be reduced to the following equation:

(31) $$\begin{array}{c} \gamma^{4}\left[1+\cosh\left(\gamma \right)\cos\left(\gamma \right)\right]+\varepsilon^{2}\delta^{2}\gamma^{8}\left[1-\cosh\left(\gamma \right)\cos\left(\gamma \right)\right]\\ -\varepsilon \left(\delta^{2}\gamma^{6}+\gamma^{4}\right)\gamma \cosh\left(\gamma \right)\sin\left(\gamma \right)-\varepsilon \left(\delta^{2}\gamma^{6}-\gamma^{4}\right)\gamma \sinh\left(\gamma \right)\cos\left(\gamma \right)=0. \end{array}$$

The above frequency equation can also be obtained from the result of Wu et al. [19].

## Results and discussion

The displacement and frequency of an AFM cantilever with consideration of crack effect for nanomachining is developed as mentioned above. The cutting force in the vertical direction is assumed to be as follows:

(32) $$F_{y}\left(\tau \right)=f_{y}\left(\sin r\omega_{n}\tau +\frac{1}{3}\sin3r\omega_{n}\tau +\frac{1}{5}\sin5r\omega_{n}\tau \right).$$

**Figure 2:**
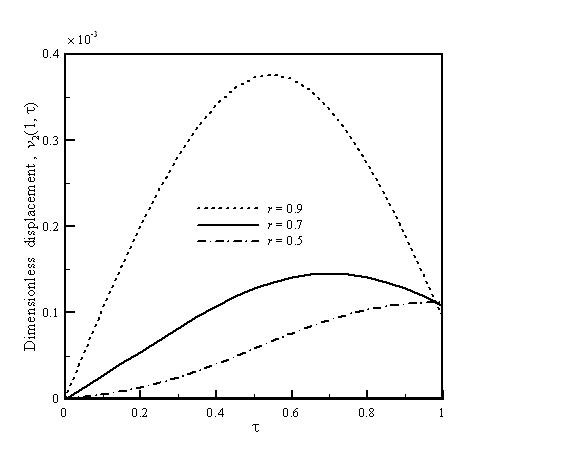
**Vibration displacement as a function of fundamental frequency ratio for the AFM nanomachining**. With *D*/*L *= 0.5, *h *= 1/30, and *k*_c _= 0.4.

During nanomachining process, the vibration displacement of the AFM cantilever is related with the cutting depth and processing quality. In this article, the effects of crack flexibility *k*_c_, crack location *D*/*L*, and tip length *h *on the displacement of the cantilever are studied. The following material properties and geometrical parameters are used [5,19]: *L *= 300 μm, *E *= 170 GPa, *I *= 33.3 × 10^-24 ^m^4^, *A *= 10^-10 ^m^2^, *H *= 10 μm, *ρ *= 2,300 kg/m^-3^, m = 2 × 10^-13 ^kg, *c *= 2.5 μm, *P *= 10^-8 ^nN, and *f*_y _= 0.16 × 10^-3^. In addition, the excitation frequency cannot be the same with the natural frequency of the cantilever. Figure 2 shows the vibration displacement which is a function of the fundamental frequency ratio, *r*, for the AFM nanomachining with *D*/*L *= 0.5, *h *= 1/30, and *k*_c _= 0.4. The value of *r *is defined as the ratio of the excitation frequency to the natural frequency of mode 1 of the cantilever. Using a different value of frequency ratio, we can obtain the different displacements of the AFM cantilever. As expected, a larger vibration displacement is obtained as the *r *value is closer to1. A larger displacement indicates a larger cutting depth.

**Figure 3:**
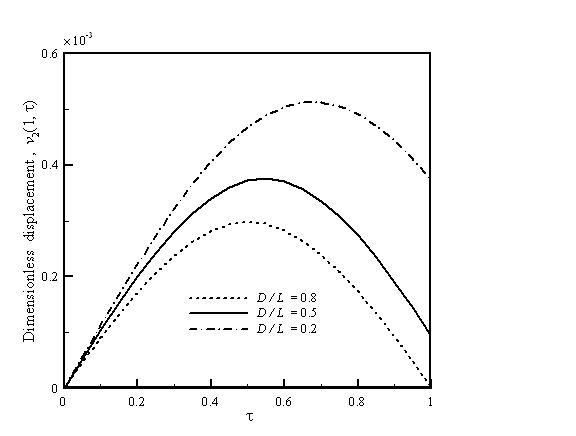
**Vibration displacement as a function of crack location for the AFM nanomachining**. With *h *= 1/30, *k*_c _= 0.4, and *r *= 0.9.

**Figure 4:**
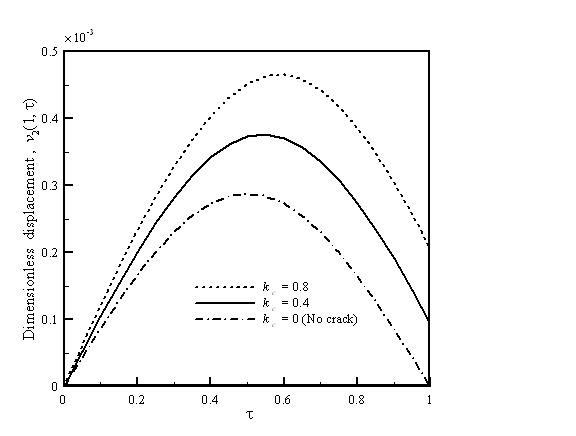
**Vibration displacement as a function of crack flexibility for the AFM nanomachining**. With *h *= 1/30, *D*/*L *= 0.5, and *r *= 0.9.

Figure 3 depicts the vibration displacement which is a function of crack location for the AFM nanomachining with *h *= 1/30, *k*_c _= 0.4, and *r *= 0.9. The cantilever has a larger flexibility as the crack is closer to the fixed end. Therefore, the cantilever shows a significant increase in the vibration displacement when the crack is near the fixed end. The crack flexibility indicates crack configuration and crack depth and is related to the changes of natural frequency and displacement of the cantilever. The vibration displacement is a function of crack flexibility for the AFM nanomachining with *h *= 1/30, *D*/*L *= 0.5, and *r *= 0.9 which is depicted in Figure 4. A crack makes the cantilever locally less stiff because of the added flexibility. Therefore, the displacement of the cracked cantilever increases with the increasing value of crack flexibility. As for the case of *k*_c _= 0, it implies no crack in the cantilever so that its displacement is the lowest.

**Figure 5:**
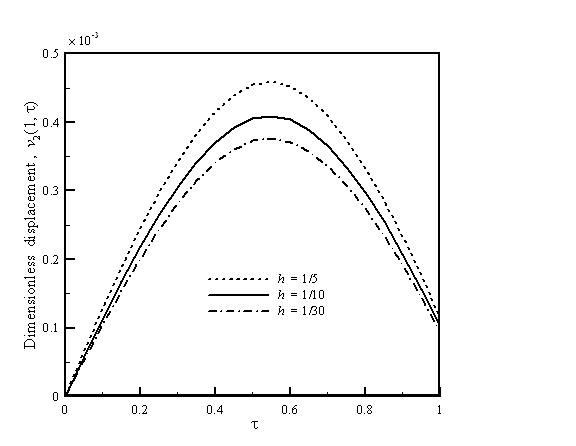
**Vibration displacement as a function of tip length for the AFM nanomachining**. With *D*/*L *= 0.5, *r *= 0.9, and *k*_c _= 0.4.

Figure 5 shows the vibration displacement which is a function of tip length for the AFM nanomachining with *D*/*L *= 0.5,*r *= 0.9, and *k*_c _= 0.4. A higher tip length results in a larger flexibility. Therefore, it can be found that a higher vibration displacement is obtained when the value of *h *is larger. Figures 2, 3, 4, and 5 can be used to predict peak displacement. The area under the curve is related to the material removal rate. For a more uniform depth of cut, the flatter curves are better. In addition, *D*/*L *ratios, *r*, and *k*_c _are important design parameters of an AFM cantilever with a crack; we can use them to control nanomachining. The change in *r *is a more effective way to control displacement than *D*/*L *or *k*_c_.

## Conclusions

During the nanomachining process, the effects of crack flexibility, crack location, and tip length on the vibration displacement of an AFM cantilever with a crack were analyzed. According to the analysis, the following results were obtained:

1. The vibration displacement of the cantilever significantly increased when the crack was near the fixed end.

2. The displacement of the cracked cantilever increased with increasing value of crack flexibility.

3. A higher vibration displacement of the cracked cantilever was obtained when the tip length was larger.

## Competing interests

The authors declare that they have no competing interests.

## Authors' contributions

HLL carried out the derivation of the equations and drawing of the figures. WJC carried out the topic selection, the writing of the manuscript, and other works about the submission. All authors read and approved the final manuscript.
